# Comparison of HIV/AIDS death estimates for 2019 between GBD 2019 and WHO mortality databases

**DOI:** 10.3389/fpubh.2025.1669277

**Published:** 2025-11-25

**Authors:** Xuemei Wei, Fang Qin, Juan Li, Ya Huang, Shaopeng Lu, Junjie Huang, Lu Wang, Jie Li, Yukai Zhang, Xiaoxiang Yang

**Affiliations:** 1 Department of Infectious Diseases in Children, The Maternal and Child Health Hospital of Guangxi Zhuang Autonomous Region, Nanning, Guangxi, China; 2 The People's Hospital of Chongzuo, Chongzuo, Guangxi, China; 3 Guangxi Key Laboratory of AIDS Prevention and Treatment, School of Public Health, Guangxi Medical University, Nanning, China

**Keywords:** GBD, WHO, HIV/AIDS, death estimates, mortality rate

## Abstract

**Background:**

HIV/AIDS death estimates serve as crucial indicators for monitoring the status of the epidemic and the progress of intervention projects. The Global Burden of Disease (GBD) 2019 study and the World Health Organization (WHO) Mortality Database offer open-access statistics on a global scale. This study compared HIV/AIDS death estimates between these two databases and explored the potential sources of discrepancies.

**Methods:**

HIV/AIDS death counts and mortality rates (per 100,000 population) for 2019 were extracted from both databases and categorized based on age, sex, and country. The absolute and standardized differences between the GBD and WHO estimates were analyzed across demographic groups and countries. Spearman’s correlation coefficients and regression plots were used to assess consistency and visualize the associations.

**Results:**

Among the 78 countries with overlapping data, GBD estimated that there were 108,668 deaths due to HIV/AIDS, while the WHO reported 48,754 deaths, yielding a difference of 59,914 deaths (76.1% of the mean estimate). Despite the overall strong correlations between the two reports (*r* > 0.80, *p* < 0.05), notable discrepancies were observed in male individuals, the 15–54 age group, and in several countries, including Thailand (23,563), Brazil (6,063), the Philippines (4,658), Ukraine (4,238), Peru (2,617), and the United States (2,325).

**Conclusion:**

Substantial differences in HIV/AIDS death estimates continue to exist in a few countries, likely due to variations in vital registration data quality, ICD coding practices, and estimation methodologies between databases. These findings highlight the need to strengthen mortality data systems and improve methodological transparency to enhance global HIV/AIDS mortality surveillance.

## 1 Introduction

The human immunodeficiency virus (HIV) is recognized as the primary cause of acquired immunodeficiency syndrome (AIDS) and has remained a global epidemic since the first documented case in 1981 (1, 2). Despite the notable decline in AIDS-related mortality attributed to the administration of antiretroviral therapy, HIV/AIDS continues to persist as one of the most lethal infectious diseases worldwide. In 2024, it is estimated that one person will die from HIV-related causes every minute globally (3). Importantly, in some countries, HIV/AIDS is responsible for a quarter of the total fatalities. By 2025, the United Nations (UN) set goals to interrupt the sustained transmission of HIV in the “95–95-95 Goals” program (4) and strengthen the HIV response in the era of Sustainable Development Goals (SDGs) (5). To monitor progress and determine the achievement of these goals globally and in different countries, the mortality rate associated with HIV infection can serve as a valuable indicator. However, the acquisition of these data presents significant challenges because of the lack of dependable and comprehensive data across countries.

Currently, three prominent international organizations, namely the GBD 2019 study conducted at the Institute for Health Metrics and Evaluation (IHME), the WHO Mortality Database, and the Joint United Nations Program on HIV/AIDS (UNAIDS), have provided global estimates of HIV/AIDS deaths. As the only precise AIDS-related death data available for 55 of these countries are in the UNAIDS database, they were excluded from this study. Both the GBD 2019 study and the WHO Mortality Database provide open-access statistics on the estimated HIV/AIDS mortality rates by country. Notably, the GBD 2019 study incorporates diverse data sources and employs statistical models (6), whereas the WHO Mortality Database relies on data obtained from national and regional death registration systems (7). Studies have indicated substantial disparities in death data for tuberculosis (8, 9), primary hepatocellular carcinoma (10)_,_ and road injuries (11) between the GBD 2019 study and data released by the WHO Mortality Database. Therefore, these two databases do not provide consistent mortality rates for various diseases.

Therefore, the objective of this study was to meticulously compare the estimates of HIV/AIDS deaths in 2019 from the GBD 2019 study and the WHO Mortality Database and investigate the potential factors that may have contributed to the observed variations at the national, regional, and sociodemographic index (SDI) levels. To the best of our knowledge, this is the first study to examine the disparities in HIV/AIDS-related death data provided by these two institutions.

## 2 Materials and methods

### 2.1 Data sources

In this study, the Data Health tool was employed to retrieve the GBD 2019 data, which can be accessed at (VizHub-GBD Results).^1^ This platform provides standardized, ready-to-use, country-level estimates and allows for reproducible data extraction across disease categories and demographic groups, ensuring methodological consistency. These variables include the number of deaths and mortality rates. Additionally, data from the WHO Mortality Database can be accessed online at http://www.who.int/tb/country/data/download/en/, which is the official WHO repository for vital registration data reported by member states. Using this source ensures that only nationally verified mortality data are included. The extracted variables included the total number of deaths and mortality rates (per 100,000 population), each disaggregated by age, sex, region, and country. No additional post-hoc reclassification was applied, except for harmonizing age categories into the three mutually available groups (0–14, 15–54, and >54 years). Sex alignment was ensured by maintaining consistent male and female classifications across both datasets.

To ensure comparability, we restricted the analysis to countries with available data in both databases and for the same reference year (2019). Countries and territories were included only if HIV/AIDS mortality data for 2019 were available in both the GBD 2019 study and the WHO Mortality Database. Records with missing, incomplete, or inconsistent entries for either total deaths or death rates were excluded to ensure comparability. In total, 126 countries or territories were excluded on this basis, and 78 countries with overlapping and complete data were retained for analysis. The full list of included and excluded countries or territories is provided in Supplementary Table 1.

### 2.2 Data analysis

This study analyzed the absolute differences in the estimates of HIV/AIDS deaths by age and sex between the two databases. Since the absolute difference in HIV/AIDS deaths might be influenced by the overall mortality burden of each country, we standardized the differences in death estimates by adjusting for each country’s absolute difference using the average number of deaths estimated by the WHO Mortality Database and the GBD 2019 study. The standardized difference was calculated using the formula (E_GBD_-E_WHO_)/((E_GBD_ + E_WHO_)/2), where E_GBD_ and E_WHO_ represent the number of HIV/AIDS deaths (or mortality rates per 100,000 population) estimated by the GBD 2019 study and the WHO Mortality Database, respectively. This standardization produced a relative metric that accounted for each country’s HIV/AIDS mortality level, allowing comparisons independent of the population size or baseline disease burden. Additionally, when the estimated number of deaths in a particular subgroup is less than five, the countries in that group are excluded from the standardized difference ranking (8). This exclusion criterion was applied to avoid statistical instability, as very small denominators can yield inflated or erratic percentage differences between sources.

The analysis included data categorized by region, namely Europe, Asia, North America, the Caribbean, and Central and South America. However, Africa and Oceania were excluded from the analysis due to the limited number of included countries. The SDI index divides the regions into five categories: low SDI (0–0.45), low-middle SDI (0.45–0.60), middle SDI (0.60–0.69), high-middle SDI (0.69–0.81), and high SDI (0.81–1.00). Since none of the included countries fall into the low SDI category, the low SDI partitions are excluded from the analysis.

We used the following methodologies to compare and analyze the variations and influencing factors in the recorded HIV/AIDS death estimates for 2019 obtained from two distinct databases:

1. We calculated the disparities in HIV/AIDS death estimates for 2019 between the GBD 2019 study and the WHO Mortality Database. We compared these estimates across various gender and age groups and analyzed the variations in gender and age-specific estimates between the two databases. Additionally, we examined the distribution of larger differences among countries, gender groups, and age groups.
2. Considering the HIV/AIDS burden in each country, we utilized standardized differences to contrast the variances in HIV/AIDS estimates from the two databases across various gender and age groups. Subsequently, the findings were compared with the results from the absolute differences to assess consistency.
3. The correlation coefficient was computed for each variable, and the regression line was plotted accordingly. It is important to note that random errors were not considered, except for correlations because all countries with GBD and WHO estimates were included. Since the WHO Mortality Database reports only point estimates without uncertainty intervals, we used central estimates from both databases to ensure methodological consistency. Consequently, the variability captured by the uncertainty intervals reported by GBD 2019 was not incorporated into our comparison.

## 3 Results

### 3.1 Characteristics of data and methodology used for death estimates from GBD 2019 and WHO mortality databases

In terms of data sources and methodologies for death estimation, GBD 2019 relies on diverse data sources and employs various modeling methods, encompassing vital registration systems, medical records, surveys, and other pertinent sources. In contrast, the WHO Mortality Database is compiled exclusively from death data submitted by member states to the WHO, without incorporating any modeling methods. Both the GBD 2019 and WHO Mortality Databases employed age groups for the included mortality data. However, the GBD 2019 utilizes 50 age groups, while the WHO Mortality Database utilizes nine age groups.

Additionally, in terms of country coverage, the GBD 2019 study covers 204 countries, whereas the WHO Mortality Database encompasses only 78 countries. Furthermore, the WHO Mortality Database assesses the quality of the data, whereas the GBD 2019 does not assess the quality. In the International Classification of Diseases (ICD) code, the GBD 2019 study draws upon data from various sources and employs statistical modeling techniques. It incorporates ICD-9 codes 042–044.9, ICD-10 codes B20–B24.9, and F02.4 to identify HIV/AIDS cases throughout the study period. Nevertheless, the WHO Mortality Database employs the updated ICD-10, which includes ICD-10 codes B20–B24 (Table 1).

**Table 1:** Characteristics of data and methodology for HIV/AIDS death number estimates from GBD 2019 and the WHO Mortality Database.

Variables	GBD estimates	WHO estimates
Data sources included for the dataset		
Data stratified by age	Yes	Yes
age groups	50	9
Data stratified by sex	Yes	Yes
population	GBD estimates	UN estimates
Included countries	204	78
Period	1990–2019	1950–2021
ICD	ICD-9: 042–044.9; ICD-10: B20–B24.9, F02.4	ICD-10: B20–B24
Quality assessment	No	Yes
Modeling methods		
Statistical/modeling methods applied	Cause of death ensemble approach, mixed-effects regression	No
Model covariates	Yes	No
Uncertainty incorporated	Yes	No

### 3.2 Global absolute differences in the number of HIV/AIDS death estimates in 2019 between GBD 2019 and WHO mortality databases

For the 78 countries included in this study, the GBD 2019 study reported 108,668 HIV/AIDS deaths, whereas the WHO Mortality Database reported only 48,754 HIV/AIDS deaths in 2019. This stark contrast resulted in an absolute difference of 59,914 deaths (a 55.13% increase in mortality compared to the WHO Mortality Database reference, or a 122.89% decrease in mortality compared to the GBD 2019 reference). We then categorized the individuals in the two databases into three groups according to their age at death: the 0–14 age group, the 15–54 age group, and the >54 years age group.

**Figure 1:**
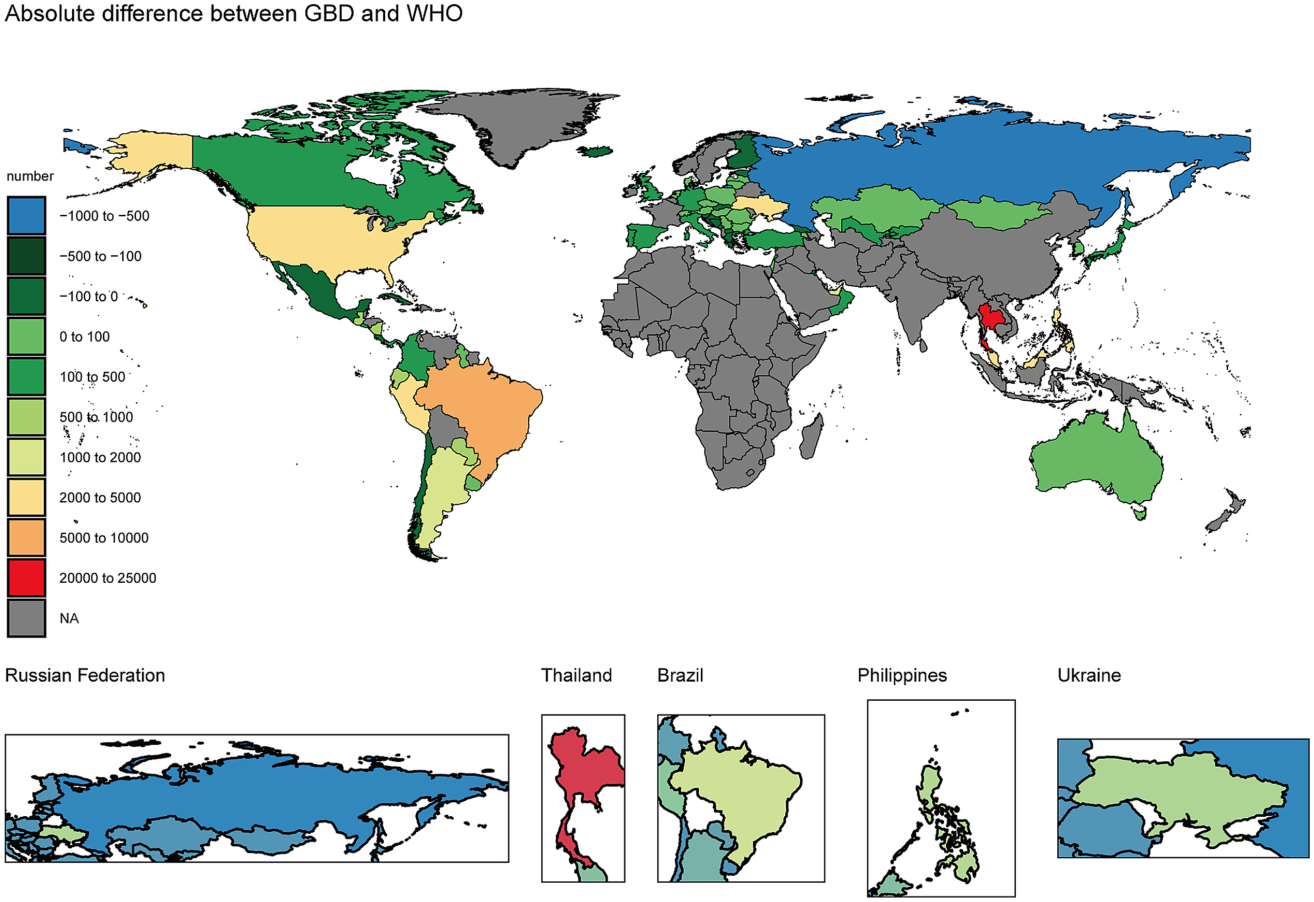
Country-level choropleth of the absolute difference in deaths (GBD − WHO). Positive values indicate GBD > WHO; negative values indicate WHO >GBD; countries without data are shown in gray. The insets show selected high-discrepancy countries (the Russian Federation, Thailand, Brazil, the Philippines, and Ukraine).

Consistent with the absence of age groups, significant differences existed in HIV/AIDS death estimates between the two databases across the three age groups. Specifically, the relative difference in mortality estimates for the children’s group (<14 years of age) was the most pronounced of the three groups, with GBD 2019 estimating 13.4 times more deaths than the WHO Mortality Database (3,910 vs. 292 deaths by GBD 2019 and the WHO Mortality Database, respectively). Similarly, there were also disparities in the number of deaths among the 15–54-year-old and the > 54-year-old groups, with GBD 2019 estimating 1.46 and 1.48 times more deaths than the WHO Mortality Database, respectively. Overall, the GBD 2019 and the WHO Mortality Databases report more than twice as many male deaths as female deaths from HIV/AIDS (with only a small difference in the 0–14 age group) (Table 2). To visualize geographic patterns in the absolute differences, we mapped the country-level (GBD-WHO) values for 2019 (Figure 1); countries without data are shown in gray.

**Table 2:** Global absolute differences in HIV/AIDS number of deaths during 2019, as estimated by GBD 2019 and the WHO Mortality Database, by sex and age group.

Age group	Sex	No. deaths (GBD)	No. deaths (WHO)	Difference	% difference (GBD ref)	% difference (WHO ref)
All ages	Both	108,668	48,754	59,914	−122.89%	55.13%
	Male	64,390	44,278	20,112	−45.42%	31.23%
	Female	30,473	18,281	12,192	−66.69%	40.01%
Ages 0–14	Both	3,910	292	3,618	−1239.04%	92.53%
	Male	2081	155	1926	−1242.58%	92.55%
	Female	1829	137	1,692	−1235.04%	92.51%
Ages 15–54	Both	74,476	51,169	23,307	−45.55%	31.29%
	Male	51,431	36,135	15,296	−42.33%	29.74%
	Female	23,045	15,034	8,011	−53.29%	34.76%
Over the age of 54	Both	16,477	11,098	5,379	−48.47%	32.65%
	Male	10,878	7,988	2,890	−36.18%	26.57%
	Female	5,599	3,110	2,489	−80.03%	44.45%

### 3.3 Countries with large differences between GBD 2019 and WHO mortality databases

**Figure 2:**
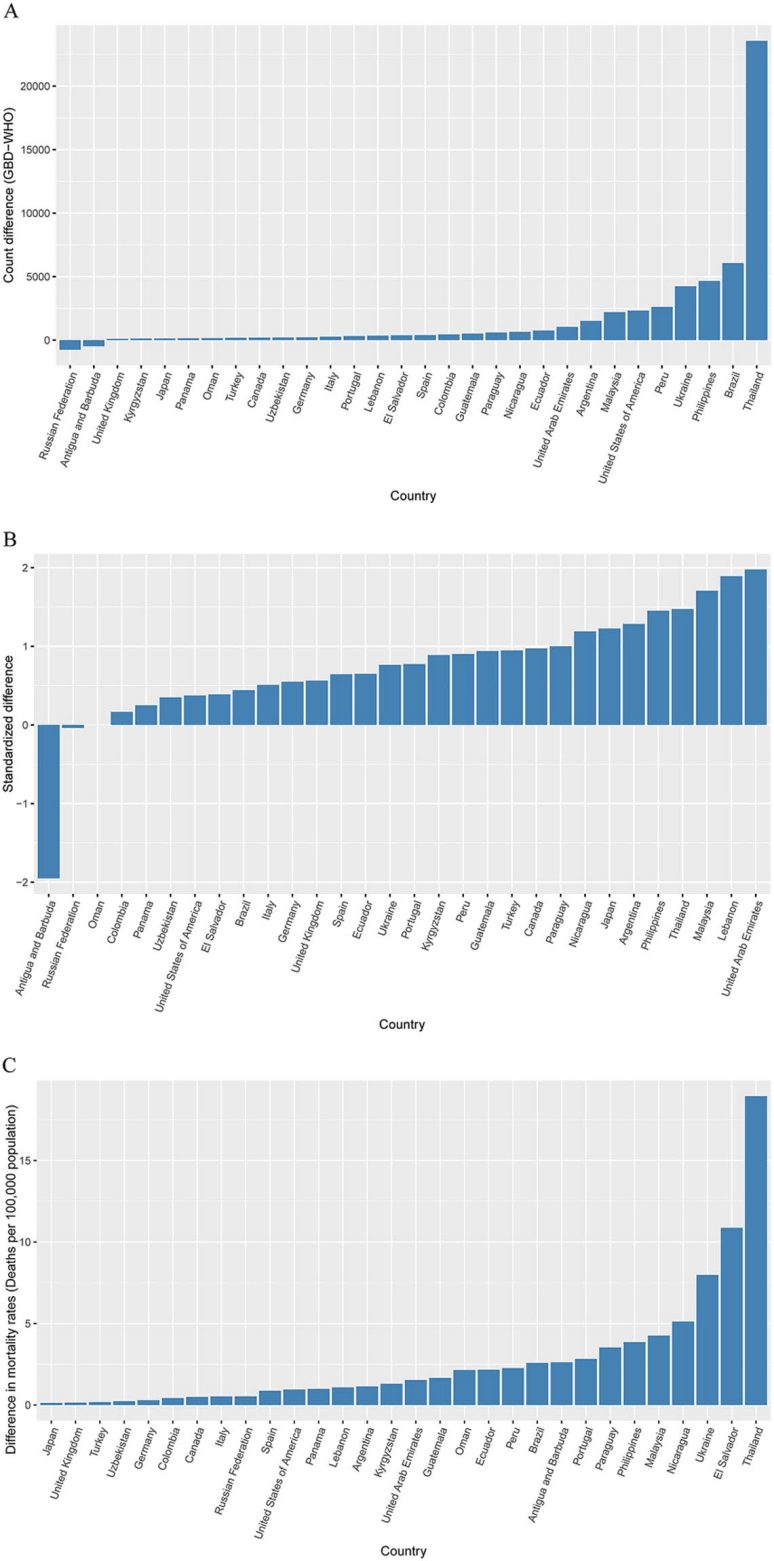
Ranking by **(A)** magnitude of absolute difference between GBD 2019 and the WHO Mortality Database, **(B)** standardized differences in the number of deaths in different countries, and **(C)** absolute difference in mortality rate (deaths per 100,000 population) of deaths in different countries.

To understand the sources of discrepancies in the estimated HIV/AIDS death values between the GBD 2019 and WHO Mortality Databases, we first grouped the countries. Among the 78 countries analyzed in this study, the disparity in HIV/AIDS death estimates between the Global Burden of Disease (GBD) 2019 and the World Health Organization (WHO) Mortality Database was below 10 in 32 countries (41.02%). Furthermore, in 45 countries (57.7%), the estimated number of deaths was less than 50. The difference was estimated to be greater than 500 in only 14 countries (14.8%) (Supplementary Table 2). The top 10 countries with the largest absolute differences in HIV/AIDS deaths were as follows: Thailand (23,563), Brazil (6,063), the Philippines (4,658), Ukraine (4,238), Peru (2,617), the United States of America (2,325), Malaysia (2,201), Argentina (1,511), the United Arab Emirates (1,043), and the Russian Federation (758) (Figure 2A). After standardization, the countries with the largest variation in estimates were as follows: the United Arab Emirates (197.73%), Lebanon (189.25%), Malaysia (170.69%), Thailand (147.42%), the Philippines (145.24%), and Antigua and Barbuda (−195.13%) (Figure 2B).

Utilizing the death rates per 100,000 (mortality rate) as a comparison indicator enables a more precise and equitable comparison of GBD 2019 and the WHO Mortality Database HIV/AIDS death estimates. This approach helps to mitigate the impact of different country population sizes, allowing for a more accurate comparison of HIV/AIDS death estimates between the two databases (12). Among the 78 countries, GBD 2019 estimated that the mortality rate (per 100,000 population) of HIV/AIDS in 2019 was higher in 65 countries (83.3%) compared to the estimate in the WHO Mortality Database. The 10 countries with the largest absolute differences in mortality rate (per 100,000), which decreased over time, were Thailand (18.95), El Salvador (10.87), Guyana (8.86), Ukraine (7.98), Dominica (7.30), Grenada (5.29), Nicaragua (5.12), Malaysia (4.26), the Philippines (3.86), and Seychelles (−11.27) (Figure 2C).

### 3.4 The differences in HIV/AIDS death estimates between GBD 2019 and WHO mortality databases by gender and age

**Figure 3:**
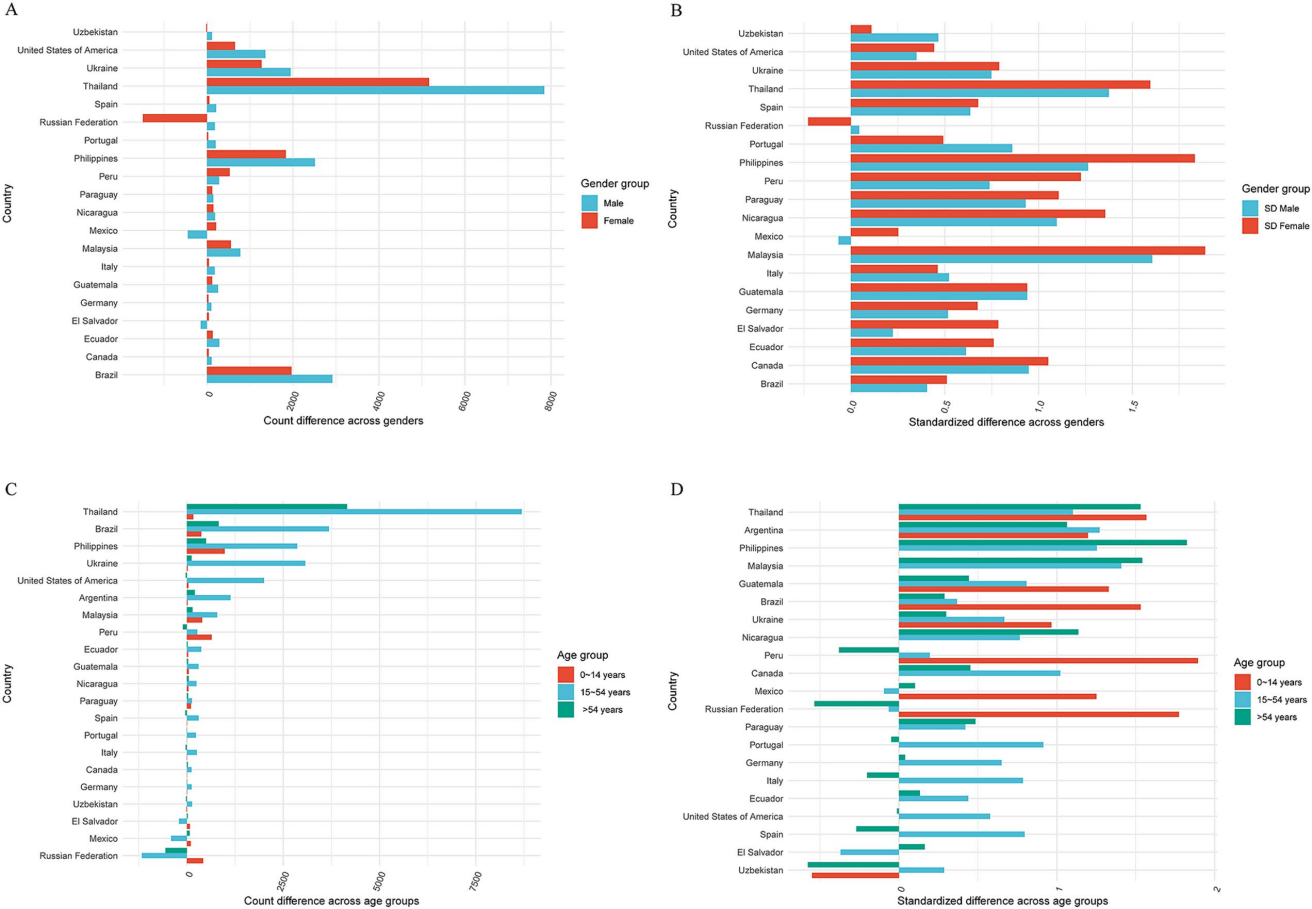
Ranking by magnitude of absolute difference of GBD 2019 and the WHO Mortality Database HIV/AIDS estimated death (all ages, gender) **(A)**; standardized difference (all ages, gender) **(B)**; HIV/AIDS estimated death (both, age group) **(C)** and standardized difference (both, age group) **(D)**.

Next, we conducted a grouped analysis of the estimated death counts by gender and age for countries in the aforementioned two databases. In countries where the disparity between the estimates from GBD 2019 and the WHO Mortality Database exceeds 100, significant gender differences in HIV/AIDS are observed (8). This threshold was used as a descriptive indicator to identify countries with substantial disparities. For instance, in the Russian Federation, the WHO Mortality Database estimates a higher number of women (1491) compared to GBD 2019, while the number of men is lower (182). Similarly, in Mexico, the WHO Mortality Database estimates a lower number of females (212) but a higher number of males (447) compared to GBD 2019. In the majority of countries (79.5%), the differences in HIV/AIDS death estimates for males were greater than those for females. Notably, Thailand (2,679) and the Russian Federation (1308) exhibited differences exceeding 1,000 cases (Figure 3A). After standardization, the countries with the largest variation in estimates of males and females were as follows: Mongolia (175.76% vs. 133.33%), Malaysia (160.56% vs. 188.77%), Bulgaria (152.00% vs. 133.33%), and Thailand (137.45% vs. 159.52%) (Figure 3B).

Among the three age groups, the difference in HIV/AIDS death estimates between the two databases was largest in the 15–54 age group. Countries with differences of more than 1,000 include Thailand (8,690), Brazil (3,686), Ukraine (3,074), the Philippines (2,864), the United States of America (2,004), Argentina (1,133), and the Russian Federation (1,171) (Figure 3C). After standardization, the order of countries with differences was as follows: Argentina (127.10%), the Philippines (125.34%), Thailand (110.18%), Brazil (36.82%), Ukraine (66.76%), the United States of America (57.76%), and the Russian Federation (6.49%) (Figure 3D).

### 3.5 Correlation of HIV/AIDS death estimates between GBD 2019 and WHO mortality databases by country and region/SDI region

**Figure 4:**
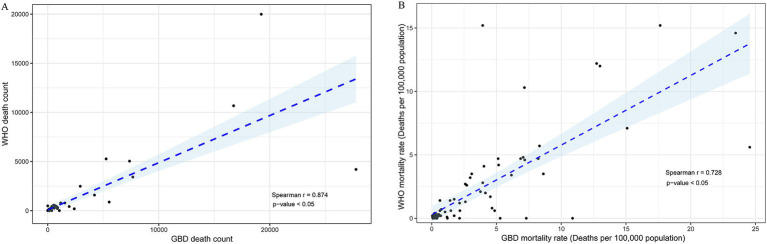
Correlations of the number of deaths **(A)** and the mortality rate (deaths per 100,000 population) **(B)** for HIV/AIDS from 78 selected countries between the GBD 2019 and the WHO Mortality Database.

**Figure 5:**
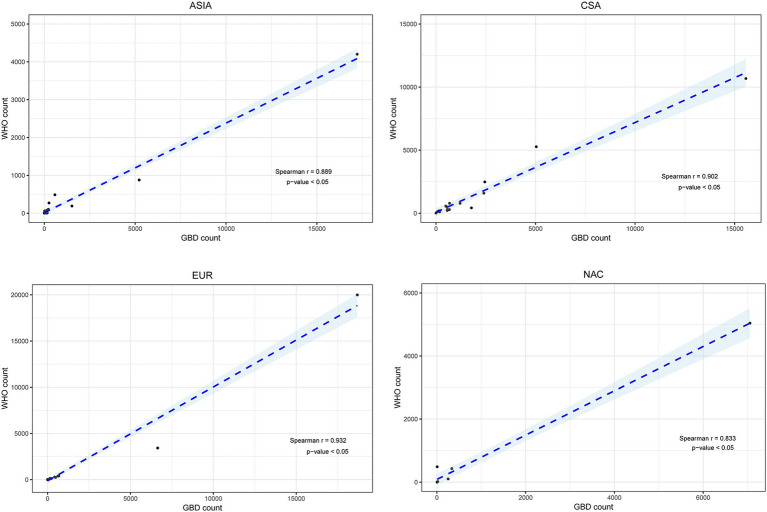
Correlations of HIV/AIDS number of deaths between GBD 2019 and the WHO Mortality Database by region.

**Figure 6:**
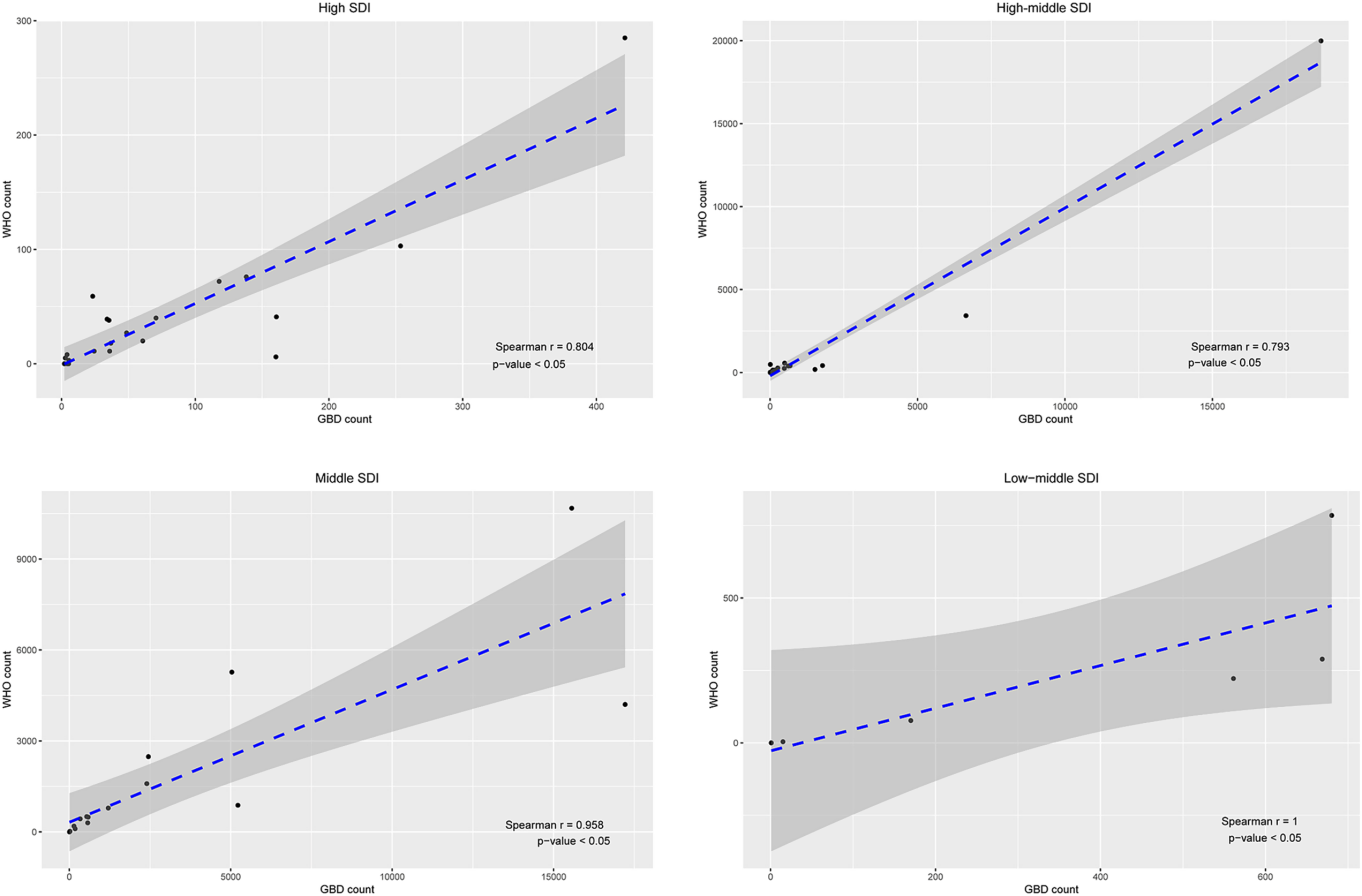
Correlations of HIV/AIDS number of deaths between GBD 2019 and WHO Mortality by socio-demographic index (SDI).

The correlation of HIV/AIDS death estimates (*r* = 0.874, *P* < 0.05) and GBD mortality rate (*r* = 0.723, *P <* 0.05) between GBD 2019 and the WHO Mortality Database was very good for 78 selected countries (Figure 4). The estimates of HIV/AIDS deaths between the two methods exhibited strong correlations within each region, divided by the location of 78 countries, with correlation coefficients (r) all greater than 0.80, except in EUR (*r* = 0.777) (Figure 5). In addition, strong correlations were also observed when the countries were redivided into four SDI regions (*r* = 1.00 for the low-middle SDI region; *r* = 0.958 for the middle SDI region; *r* = 0.793 for the high-middle SDI region; *r* = 0.804 for the high SDI region) (Figure 6).

## 4 Discussion

Accurate mortality data can reveal trends of the HIV/AIDS epidemic and the process of global intervention strategies and measures (13, 14). In this study, we compared HIV/AIDS death estimates and death rates per 100,000 population between GBD 2019 and the WHO Mortality Database. Three main findings emerged: (1) the data sources, modeling, and data processing methods of the GBD 2019 study and WHO Mortality Database are very different; (2) although the mortality estimates in the majority of countries were similar between the two databases, a few countries exhibited very large differences, even more than 1,000 deaths; and (3) after adjusting for differences in population size for countries by death rates per 100,000 (mortality rate), the disparities of death estimates in most countries have decreased. These discrepancies can be broadly attributed to differences in data quality and completeness, ART coverage, and methodological variations in modeling and estimation approaches.

First, the sources of HIV/AIDS mortality estimates for the WHO Mortality Database and GBD 2019 are fundamentally different. The WHO Mortality Database publishes cause-of-death statistics based on the national civil registration and vital statistics (VR) system data submitted by its member states, excluding lay diagnoses of the causes of death (15). However, the adoption of health estimates by the WHO Mortality Database may be influenced by various factors (16), including country consultation processes that incorporate country-level health estimates, existing collaboration mechanisms with multiple agencies and expert groups, and adherence to standards for reporting data and methods. For example, the vital registration system in India is still in its developmental stage (17). In addition, because of the impact of political instability and armed conflict, the vital registration systems of some countries have been damaged, such as Syria (18). Some countries, although possessing relevant data, have declined to share them with the WHO Mortality Database because of concerns regarding data misuse or misinterpretation (19). These issues represent data quality and reporting limitations that may lead to underreporting and incomplete mortality records in the WHO data.

Second, differences in ART coverage and treatment program scale-up may influence how deaths are classified and captured. In settings where ART access is widespread, improved survival and lower AIDS-related mortality may lead to underrepresentation in cause-of-death reporting (20), whereas in countries with limited ART availability, HIV-related deaths may be more prominent but inconsistently recorded (21). These programmatic disparities may partly explain the heterogeneity between the two databases in countries such as Thailand (22), Brazil (23), and the Philippines (21), where treatment expansion and surveillance capacities differ markedly.

Third, methodological and modeling differences also play a major role in shaping discrepancies. GBD 2019 employs a covariate-based estimation framework developed by the IHME to refine health estimates derived from the WHO’s global dataset from 2000 to 2019 (16). The estimates are synthesized from multiple data sources, including censuses, surveys, disease registries, and health service utilization records. In contrast, WHO estimates are grounded in officially reported VR data, which may be influenced by country consultation processes, multi-agency coordination, and adherence to specific reporting standards (15). Moreover, while the WHO Mortality Database has technical authority in public health, it lacks sufficient legal leverage to influence reporting from non-cooperative countries (18). These distinctions reflect methodological modeling differences, where GBD 2019 employs methods to reassign garbage-coded deaths in vital registration data for more accurate allocation.

Furthermore, the identification and coding of HIV pose additional challenges because of stigma, confidentiality issues, and symptom overlap with other diseases (24), which can lead to significant under-reporting and data inaccuracies.

This study has several limitations. First, the analysis included only 78 countries with overlapping data, excluding many African nations with the highest HIV/AIDS burden. This restricted coverage may bias global interpretations and underestimate the true magnitude of the discrepancies. Second, to ensure methodological comparability, only central estimates were analyzed, and no formal model validation or uncertainty quantification was performed. This limitation may affect the statistical robustness of the findings. Third, the correlation analysis assumed independence among variables and did not explicitly account for random errors; the study was cross-sectional for a single year (2019); therefore, temporal variability could not be evaluated.

## 5 Conclusion

In summary, there is strong agreement between the GBD 2019 study and the WHO Mortality Database estimates of HIV/AIDS deaths and mortality rates across the 78 countries analyzed, although notable inconsistencies continue to remain in some settings. These discrepancies likely reflect variations in data quality, cause-of-death classification, and estimation methodologies. Therefore, caution is needed when interpreting national-level estimates for policy and monitoring purposes. Future research should focus on integrating multiple data sources, improving transparency in modeling assumptions, and strengthening the data linkage between national and global systems. In addition, access to detailed input data and modeling information should be enhanced to clarify the sources of database discrepancies and improve the completeness and reliability of global HIV/AIDS mortality assessments.

## Data Availability

Publicly available datasets were analyzed in this study. This data can be found here: The HIV/AIDS data analyzed in this study are derived from publicly available datasets: The Global Burden of Disease (GBD) 2019 data can be found at: https://ghdx.healthdata.org/gbd-2019. World Health Organization Mortality Database can be found at: https://platform.who.int/mortality/themes/theme-details/topics/indicator-groups/indicator-group-details/MDB/hiv-aids.

## Footnotes

1
healthdata.org
