# Supplementary material for: Comparison of HIV/AIDS death estimates for 2019 between GBD 2019 and WHO mortality databases
Source: Front Public Health. 2025 Nov 25;13:1669277. doi: 10.3389/fpubh.2025.1669277 (PMC12685798; doi:10.3389/fpubh.2025.1669277)
Supplement: Supplementary file 1 [file Table_1.docx]

| Supplementary Table 1 List of retained or excluded locations | |
| --- | --- |
| Retained location (n = 78) | Excluded locations(n = 126) |
| Antigua and Barbuda | China |
| Argentina | Taiwan (Province of China) |
| Armenia | Democratic People's Republic of Korea |
| Australia | Cambodia |
| Austria | Lao People's Democratic Republic |
| Bosnia and Herzegovina | Indonesia |
| Brazil | Myanmar |
| Brunei Darussalam | Sri Lanka |
| Bulgaria | Fiji |
| Canada | Micronesia (Federated States of) |
| Chile | Timor-Leste |
| Colombia | Samoa |
| Costa Rica | Viet Nam |
| Croatia | Papua New Guinea |
| Cuba | Kiribati |
| Cyprus | Marshall Islands |
| Czechia | Tonga |
| Denmark | Vanuatu |
| Dominica | Solomon Islands |
| Ecuador | Azerbaijan |
| El Salvador | Tajikistan |
| Estonia | Turkmenistan |
| Finland | Albania |
| Georgia | Belarus |
| Germany | Republic of Moldova |
| Greece | New Zealand |
| Grenada | Andorra |
| Guatemala | Belgium |
| Guyana | Malta |
| Hungary | France |
| Iceland | Ireland |
| Israel | Norway |
| Italy | Sweden |
| Japan | Barbados |
| Kazakhstan | Belize |
| Kuwait | Bahamas |
| Kyrgyzstan | Dominican Republic |
| Latvia | Haiti |
| Lebanon | Jamaica |
| Lithuania | Suriname |
| Luxembourg | Trinidad and Tobago |
| Malaysia | Bolivia (Plurinational State of) |
| Maldives | Honduras |
| Mauritius | Venezuela (Bolivarian Republic of) |
| Mexico | Tunisia |
| Mongolia | Algeria |
| Montenegro | Bahrain |
| Netherlands | Egypt |
| Nicaragua | Iraq |
| North Macedonia | Iran (Islamic Republic of) |
| Oman | Afghanistan |
| Panama | Jordan |
| Paraguay | Palestine |
| Peru | Libya |
| Philippines | Morocco |
| Poland | Saudi Arabia |
| Portugal | Syrian Arab Republic |
| Qatar | Gabon |
| Republic of Korea | Yemen |
| Romania | India |
| Russian Federation | Bangladesh |
| Saint Lucia | Bhutan |
| Saint Vincent and the Grenadines | Nepal |
| Serbia | Pakistan |
| Seychelles | Angola |
| Singapore | Congo |
| Slovakia | Central African Republic |
| Slovenia | Democratic Republic of the Congo |
| Spain | Equatorial Guinea |
| Switzerland | Burundi |
| Thailand | Comoros |
| Turkey | Djibouti |
| Ukraine | Eritrea |
| United Arab Emirates | United Republic of Tanzania |
| United Kingdom | Ethiopia |
| United States of America | Madagascar |
| Uruguay | Kenya |
| Uzbekistan | Malawi |
|  | Mozambique |
|  | Somalia |
|  | Rwanda |
|  | Uganda |
|  | Botswana |
|  | Zambia |
|  | Lesotho |
|  | South Africa |
|  | Namibia |
|  | Eswatini |
|  | Zimbabwe |
|  | Benin |
|  | Burkina Faso |
|  | Cameroon |
|  | Chad |
|  | Nigeria |
|  | Cabo Verde |
|  | Gambia |
|  | Côte d'Ivoire |
|  | Ghana |
|  | Guinea-Bissau |
|  | Liberia |
|  | Guinea |
|  | Mali |
|  | Sao Tome and Principe |
|  | Senegal |
|  | Niger |
|  | Mauritania |
|  | Sierra Leone |
|  | Togo |
|  | Bermuda |
|  | Cook Islands |
|  | Greenland |
|  | American Samoa |
|  | Monaco |
|  | Niue |
|  | Guam |
|  | Nauru |
|  | Northern Mariana Islands |
|  | Palau |
|  | San Marino |
|  | Puerto Rico |
|  | Saint Kitts and Nevis |
|  | Tokelau |
|  | Tuvalu |
|  | United States Virgin Islands |
|  | South Sudan |
|  | Sudan |
