# Supplementary material for: Comparison of HIV/AIDS death estimates for 2019 between GBD 2019 and WHO mortality databases
Source: Front Public Health. 2025 Nov 25;13:1669277. doi: 10.3389/fpubh.2025.1669277 (PMC12685798; doi:10.3389/fpubh.2025.1669277)
Supplement: Supplementary file 2 [file Table_2.docx]

Supplementary Table 2 Absolute differences in HIV/AIDS number of deaths and standardized differences by country, age and sex.

| location | Absolute difference in number of deaths ( all ages) | Standardized difference ( all ages) | Absolute difference in number of deaths in Male ( all ages) | Standardized difference in Male ( all ages) | Absolute difference in number of deaths in Female ( all ages) | Standardized difference in Female ( all ages) | Absolute difference in number of deaths in 0 to 14 age group | Standardized difference in 0 to 14 age group | Absolute difference in number of deaths in 15 to 54 age group | Standardized difference in 15 to 54 age group | Absolute difference in number of over 54 age group | Standardized difference in over 54 age group |
| --- | --- | --- | --- | --- | --- | --- | --- | --- | --- | --- | --- | --- |
| Antigua and Barbuda | -481 | -195.13% | -301 | -192.31% | -179 | - | -30 | - | 397 | -194.52% | -52 | - |
| Argentina | 1511 | 128.43% | 822 | 105.40% | 534 | - | 18 | 119.73% | -1133 | 127.10% | 205 | 106.43% |
| Armenia | 2 | 10.00% | -1 | 6.06% | 1 | - | 0 | - | 0 | 3.03% | -1 | - |
| Australia | 38 | 64.41% | 19 | 51.55% | 12 | - | 1 | - | -30 | 93.56% | 0 | -2.13% |
| Austria | 4 | 10.00% | -2 | 9.52% | 0 | 11.76% | 1 | - | -9 | 41.98% | -12 | -81.66% |
| Bosnia and Herzegovina | -7 | -107.69% | -9 | - | 1 | - | 0 | - | 0 | - | -8 | - |
| Brazil | 6063 | 44.23% | 2919 | 40.58% | 1966 | 51.11% | 373 | 153.16% | -3686 | 36.82% | 826 | 28.83% |
| Brunei Darussalam | 5 | 200.00% | 4 | - | 2 | - | 0 | - | -4 | - | 1 | - |
| Bulgaria | 44 | 133.33% | 30 | 152.00% | 5 | - | 1 | - | -23 | 107.38% | 11 | - |
| Canada | 195 | 97.26% | 109 | 94.74% | 41 | 105.15% | 3 | - | -121 | 102.28% | 26 | 45.27% |
| Chile | -32 | -5.82% | -30 | 0.91% | -44 | -33.03% | 1 | - | 24 | -6.06% | -51 | -38.89% |
| Colombia | 450 | 16.63% | -83 | 13.22% | 52 | 27.47% | 41 | 154.38% | 10 | -0.53% | -62 | -12.13% |
| Costa Rica | -14 | -7.65% | -54 | -20.41% | 10 | 44.44% | 6 | - | 29 | -23.03% | -22 | -55.02% |
| Croatia | -3 | -35.29% | -2 | -13.33% | 0 | - | 0 | - | 3 | - | 0 | - |
| Cuba | -65 | -16.35% | -66 | -12.69% | -27 | -34.59% | 4 | - | 54 | -18.27% | -42 | -49.60% |
| Cyprus | 4 | 200.00% | 4 | - | 1 | - | 0 | - | -3 | - | 2 | - |
| Czechia | 11 | 88.00% | 6 | 66.67% | 6 | - | 1 | - | -5 | 57.85% | 6 | - |
| Denmark | 18 | 90.00% | 8 | 66.67% | 6 | - | 0 | - | -11 | - | 2 | 19.67% |
| Dominica | 1 | 28.57% | 1 | - | 1 | - | 0 | - | -2 | - | 0 | - |
| Ecuador | 756 | 64.95% | 288 | 61.37% | 133 | 76.11% | 32 | - | -372 | 43.94% | 17 | 13.25% |
| El Salvador | 379 | 38.89% | -148 | 22.38% | 43 | 78.53% | 80 | - | 206 | -36.98% | 22 | 16.35% |
| Estonia | -1 | -2.60% | -2 | 6.67% | -3 | -35.29% | 0 | - | 2 | -4.66% | -4 | - |
| Finland | 0 | 0.00% | 2 | - | 0 | - | 0 | - | -2 | - | 0 | - |
| Georgia | -32 | -74.42% | -26 | -75.00% | -10 | -72.73% | 0 | - | 26 | -81.25% | -10 | - |
| Germany | 216 | 54.96% | 101 | 51.76% | 35 | 67.50% | 7 | - | -123 | 65.05% | 6 | 3.89% |
| Greece | -5 | -18.18% | -6 | -18.18% | -2 | - | 0 | - | 4 | -19.20% | -3 | - |
| Grenada | 4 | 200.00% | 4 | - | 1 | - | 0 | - | -4 | - | 1 | - |
| Guatemala | 512 | 93.94% | 259 | 93.94% | 121 | 93.95% | 48 | 132.90% | -301 | 80.79% | 31 | 44.34% |
| Guyana | 92 | 61.33% | 45 | 61.36% | 32 | 61.29% | 2 | - | -72 | 60.76% | 4 | 17.23% |
| Hungary | 27 | 98.18% | 19 | 104.35% | 4 | - | 0 | - | -10 | 54.09% | 13 | - |
| Iceland | -1 | -200.00% | 0 | - | 0 | - | 0 | - | -1 | - | -1 | - |
| Israel | 21 | 64.62% | 10 | 63.83% | 5 | 66.67% | 3 | - | -13 | 77.61% | -1 | -10.08% |
| Italy | 269 | 50.90% | 181 | 52.27% | 46 | 46.28% | 5 | - | -258 | 78.58% | -36 | -20.20% |
| Japan | 130 | 122.64% | 77 | 103.03% | 42 | - | 7 | - | -23 | 69.50% | 90 | 140.42% |
| Kazakhstan | 41 | 14.07% | 3 | 16.92% | -13 | 7.73% | 4 | - | 3 | -1.34% | -11 | -75.36% |
| Kuwait | -5 | -200.00% | -3 | - | 0 | - | 1 | - | 2 | - | -1 | - |
| Kyrgyzstan | 123 | 88.81% | 67 | 91.89% | 26 | 82.61% | 16 | - | -69 | 64.86% | 8 | - |
| Latvia | 65 | 62.20% | 15 | 43.33% | 30 | 87.64% | 1 | - | -47 | 60.66% | -2 | -10.59% |
| Lebanon | 352 | 189.25% | 25 | 185.12% | 26 | - | 16 | - | -29 | 141.59% | 6 | - |
| Lithuania | 51 | 112.09% | 6 | 50.00% | 35 | - | 0 | - | -30 | 87.62% | 11 | - |
| Luxembourg | 0 | #DIV/0! | 2 | - | 1 | - | 0 | - | -2 | - | 0 | - |
| Malaysia | 2201 | 170.69% | 775 | 160.56% | 557 | 188.77% | 399 | - | -785 | 140.81% | 148 | 154.17% |
| Maldives | 0 | #DIV/0! | 0 | - | 1 | - | 0 | - | -1 | - | 0 | - |
| Mauritius | -36 | -27.91% | -38 | -20.10% | -17 | -61.22% | 0 | - | 41 | -41.46% | -15 | -71.77% |
| Mexico | -10 | -0.19% | -447 | -6.61% | 212 | 25.28% | 100 | 125.17% | 410 | -9.46% | 75 | 10.16% |
| Mongolia | 37 | 164.44% | 10 | - | 1 | - | 1 | - | -6 | - | 4 | - |
| Montenegro | -2 | -200.00% | -1 | - | 0 | - | 0 | - | 1 | - | 0 | - |
| Netherlands | 29 | 69.88% | 12 | 53.97% | 10 | - | 1 | - | -17 | 74.88% | 4 | 24.63% |
| Nicaragua | 651 | 118.90% | 189 | 109.66% | 150 | 135.55% | 43 | - | -249 | 76.53% | 47 | 113.66% |
| North Macedonia | 0 | #DIV/0! | 1 | - | 1 | - | 1 | - | -1 | - | 0 | - |
| Oman | 157 | 195.03% | 79 | - | 17 | - | 2 | - | -81 | - | 13 | - |
| Panama | 144 | 25.00% | 32 | 26.07% | 5 | 21.75% | 0 | 3.91% | -54 | 12.71% | -18 | -21.06% |
| Paraguay | 598 | 100.00% | 148 | 93.15% | 123 | 110.73% | 108 | - | -131 | 42.09% | 33 | 48.49% |
| Peru | 2617 | 90.26% | 281 | 73.88% | 529 | 122.54% | 645 | 189.42% | -270 | 19.53% | -105 | -38.03% |
| Philippines | 4658 | 145.24% | 2511 | 126.38% | 1834 | 183.26% | 983 | - | -2864 | 125.34% | 498 | 182.41% |
| Poland | 46 | 38.33% | 24 | 31.58% | 13 | 64.00% | 5 | - | -29 | 30.44% | 4 | 19.96% |
| Portugal | 317 | 77.41% | 202 | 85.99% | 29 | 49.21% | 2 | - | -235 | 91.54% | -5 | -4.90% |
| Qatar | 4 | 200.00% | 3 | - | 1 | - | 0 | - | -4 | - | 0 | - |
| Republic of Korea | 95 | 76.92% | 54 | 75.56% | 8 | 90.91% | 3 | - | -35 | 66.88% | 24 | 45.65% |
| Romania | 36 | 23.38% | 1 | 18.67% | 5 | 36.14% | 6 | - | 8 | -6.36% | 8 | 43.50% |
| Russian Federation | -758 | -3.87% | 182 | 4.44% | -1491 | -22.79% | 423 | 177.34% | 1171 | -6.49% | -560 | -53.64% |
| Saint Lucia | 1 | 22.22% | 4 | - | 0 | - | 0 | - | -2 | - | 1 | - |
| Saint Vincent and the Grenadines | 2 | 10.53% | -1 | 0.00% | 3 | - | 0 | - | -1 | 7.44% | 0 | - |
| Serbia | 90 | 128.57% | 18 | 129.20% | 5 | - | 4 | - | -22 | 96.34% | -3 | -29.31% |
| Seychelles | -15 | -200.00% | -8 | - | -3 | - | -1 | - | 5 | - | -5 | - |
| Singapore | 34 | 121.43% | 24 | 128.00% | 1 | - | 0 | - | -17 | 116.28% | 8 | - |
| Slovakia | -5 | -90.91% | -5 | - | 1 | - | 0 | - | 2 | - | -2 | - |
| Slovenia | 0 | #DIV/0! | 2 | - | 0 | - | 0 | - | -2 | - | 0 | - |
| Spain | 391 | 64.47% | 212 | 63.66% | 56 | 67.78% | 7 | - | -304 | 79.62% | -43 | -27.04% |
| Switzerland | 25 | 81.97% | 12 | 66.67% | 7 | - | 1 | - | -19 | 114.38% | 0 | -2.85% |
| Thailand | 23563 | 147.42% | 7846 | 137.46% | 5167 | 159.52% | 167 | 156.81% | -8690 | 110.18% | 4156 | 153.07% |
| Turkey | 185 | 94.63% | 64 | 75.91% | 59 | 138.46% | 20 | - | -87 | 79.58% | 16 | 37.66% |
| Ukraine | 4238 | 76.43% | 1946 | 74.90% | 1271 | 79.05% | 21 | 96.65% | -3074 | 66.76% | 122 | 29.92% |
| United Arab Emirates | 1043 | 197.73% | 138 | - | 17 | - | 2 | - | -116 | - | 36 | - |
| United Kingdom | 106 | 56.38% | 43 | 35.29% | 55 | 111.54% | 7 | - | -83 | 68.41% | 7 | 12.14% |
| United States of America | 2325 | 37.47% | 1360 | 34.96% | 651 | 44.36% | 40 | - | -2004 | 57.76% | -33 | -1.31% |
| Uruguay | 39 | 22.48% | 31 | 33.85% | -8 | -11.49% | 1 | - | -3 | 2.52% | 20 | 38.69% |
| Uzbekistan | 207 | 35.06% | 116 | 46.62% | -19 | 10.97% | -13 | -55.15% | -135 | 28.65% | -24 | -57.79% |
